# Neurometabolic topography and associations with cognition in Alzheimer's disease: A whole‐brain high‐resolution 3D MRSI study

**DOI:** 10.1002/alz.14137

**Published:** 2024-07-29

**Authors:** Jialin Hu, Miao Zhang, Yaoyu Zhang, Huixiang Zhuang, Yibo Zhao, Yudu Li, Wen Jin, Xiao‐Hang Qian, Lijun Wang, Guanyu Ye, Huidong Tang, Jun Liu, Biao Li, Parashkev Nachev, Zhi‐Pei Liang, Yao Li

**Affiliations:** ^1^ National Engineering Research Center of Advanced Magnetic Resonance Technologies for Diagnosis and Therapy, School of Biomedical Engineering Shanghai Jiao Tong University Shanghai China; ^2^ Department of Nuclear Medicine Ruijin Hospital Shanghai Jiao Tong University School of Medicine Shanghai China; ^3^ Beckman Institute for Advanced Science and Technology University of Illinois at Urbana‐Champaign Urbana Illinois USA; ^4^ Department of Electrical and Computer Engineering University of Illinois at Urbana‐Champaign Urbana Illinois USA; ^5^ National Center for Supercomputing Applications University of Illinois at Urbana‐Champaign Urbana Illinois USA; ^6^ Department of Geriatrics Ruijin Hospital Shanghai Jiao Tong University School of Medicine Shanghai China; ^7^ Medical Center on Aging of Ruijin Hospital Shanghai Jiao Tong University School of Medicine Shanghai China; ^8^ Department of Neurology and Institute of Neurology Ruijin Hospital Shanghai Jiao Tong University School of Medicine Shanghai China; ^9^ Department of Neurovascular Center Changhai Hospital Naval Medical University Shanghai China; ^10^ High‐Dimensional Neurology Group Institute of Neurology University College London London UK; ^11^ Institute of Medical Robotics Shanghai Jiao Tong University Shanghai China

**Keywords:** Alzheimer's disease, cognition, magnetic resonance spectroscopic imaging, myo‐inositol, N‐acetylaspartate

## Abstract

**INTRODUCTION:**

Altered neurometabolism, detectable via proton magnetic resonance spectroscopic imaging (^1^H‐MRSI), is spatially heterogeneous and underpins cognitive impairments in Alzheimer's disease (AD). However, the spatial relationships between neurometabolic topography and cognitive impairment in AD remain unexplored due to technical limitations.

**METHODS:**

We used a novel whole‐brain high‐resolution ^1^H‐MRSI technique, with simultaneously acquired ^18^F‐florbetapir positron emission tomography (PET) imaging, to investigate the relationship between neurometabolic topography and cognitive functions in 117 participants, including 22 prodromal AD, 51 AD dementia, and 44 controls.

**RESULTS:**

Prodromal AD and AD dementia patients exhibited spatially distinct reductions in N‐acetylaspartate, and increases in myo‐inositol. Reduced N‐acetylaspartate and increased myo‐inositol were associated with worse global cognitive performance, and N‐acetylaspartate correlated with five specific cognitive scores. Neurometabolic topography provides biological insights into diverse cognitive dysfunctions.

**DISCUSSION:**

Whole‐brain high‐resolution ^1^H‐MRSI revealed spatially distinct neurometabolic topographies associated with cognitive decline in AD, suggesting potential for noninvasive brain metabolic imaging to track AD progression.

**Highlights:**

- Whole‐brain high‐resolution ^1^H‐MRSI unveils neurometabolic topography in AD.
- Spatially distinct reductions in NAA, and increases in mI, are demonstrated.
- NAA and mI topography correlates with global cognitive performance.
- NAA topography correlates with specific cognitive performance.

## 1 BACKGROUND

Alzheimer's disease (AD) is characterized by a progressive decline in cognition and behavior. Initial cognitive deficits typically manifest as compromised episodic memory, attributable to neurochemical and pathological changes in the medial temporal lobe. As AD progresses—and in some variants at the outset—impairments extend to language, attention, visuospatial, and executive functions due to wider cortical dysfunction, in patterns varying across the population.^1^, ^2^ The pathogenesis of AD is complex, involving disruption in neuronal function associated with the development of neurofibrillary tangles and neuritic plaques, primarily within cortical and medial temporal limbic regions, but varying anatomically across individuals. These spatially heterogeneous patterns plausibly result in correspondingly heterogeneous downstream neurometabolic changes, reflecting the diverse manifestations of cognitive impairment observed in AD.

Proton magnetic resonance spectroscopic imaging (^1^H‐MRSI) offers a potentially powerful tool for noninvasive mapping of neurometabolite changes associated with AD, without the need for exogenous contrast agents or radioactive tracers. Previous magnetic resonance spectroscopy (MRS) studies in AD have unveiled changes in regional metabolite concentrations, particularly a decline in N‐acetylaspartate (NAA) indicative of neuronal loss or mitochondrial dysfunction, and an elevation in myo‐inositol (mI) associated with gliosis.^3^, ^4^, ^5^, ^6^, ^7^, ^8^, ^9^, ^10^ Reduced NAA and increased mI levels have shown correlations with increased disease severity and diminished cognitive function, with specific brain regions exhibiting varying correlations across distinct cognitive domains.^5^, ^11^, ^12^, ^13^, ^14^ However, prior MRS studies in AD provide limited insights into topographical details of neurometabolites, primarily due to the use of single‐voxel MRS, which measures metabolites from only one or very few regions of interest at a time. Despite efforts to explore neurometabolic alterations using MRSI technology, its ability for precise measurements of global and region‐specific neurometabolite concentrations has been limited by low spatial resolution (over 20 × 20 × 20 mm^3^ for a single voxel and over 10 mm for a single slice for 2D imaging), long acquisition time (over 5 min for a single slice), and incomplete brain coverage.^3^, ^7^, ^15^, ^16^, ^17^, ^18^, ^19^ Hence, the in vivo establishment of spatial relationships between neurometabolic topography and cognitive impairments in AD remains an unmet challenge.

In this study, we employed a novel 3D ^1^H‐MRSI technique, namely SPICE (SPectroscopic Imaging by exploiting spatiospectral correlation), to accomplish whole‐brain high‐resolution (2 × 3 × 3 mm^3^) mapping of neurometabolites in less than 10 min of scanning time.^20^, ^21^, ^22^, ^23^, ^24^, ^25^, ^26^ Leveraging the capability for whole‐brain imaging, we investigated the relationship between neurometabolic topographies and clinical status, as well as, neuropsychological performance in individuals with prodromal AD and AD dementia. The high‐resolution imaging capability facilitates the precise determination of region‐specific metabolite concentrations, mitigating the impact of partial volume effects. Our objective was to identify brain regions where NAA reduction and mI increase are most strongly associated with cognitive features of AD. Associations were established between neurometabolic variations and specific neuropsychological performances, including verbal episodic memory, visual episodic memory, attention/processing speed, and visuospatial abilities. Furthermore, we assessed the extent to which neurometabolic topography, measured by 3D ^1^H‐MRSI, could predict cognitive decline in different cognitive domains. Neurometabolic topography, revealed by whole‐brain metabolic imaging, characterizes spatially distinct distributions of neurometabolic patterns, providing a comprehensive understanding of spatially related neurometabolic changes in AD.

## 2 METHODS

### 2.1 Participants

A total of 117 participants, recruited from the aging study at Memory Clinic of Ruijin Hospital between January 2019 and November 2021, were enrolled in this study. This cohort comprised 44 amyloid‐β (Aβ) negative cognitively normal (CN) individuals, 22 Aβ positive individuals with amnestic mild cognitive impairment (aMCI), and 51 Aβ‐positive patients with AD. The Aβ status was determined by ^18^F‐florbetapir PET imaging following the procedure in previous literature.^27^, ^28^ All participants underwent clinical assessments with Clinical Dementia Rating (CDR)^29^ and Mini‐Mental State Examination (MMSE) tests.^30^ Patients with AD dementia were further categorized into mild AD (CDR = 1) and moderate AD (CDR = 2) groups based on the global CDR score. All groups were matched for age and sex (Table 1). A consensus diagnosis of aMCI was made according to the Petersen criteria,^31^ and the clinical diagnosis of AD was made based on the NIA‐AA criteria,^32^ by a panel of experienced neurologists. Exclusion criteria included: (1) psychiatric or other neurological diseases; (2) pregnancy or renal failure; (3) major systemic disease; (4) history of traumatic brain injury; and (5) history of drug or alcohol addiction. The study was approved by the Institutional Review Board of Ruijin Hospital, in accordance with the Helsinki Declaration and its later revised ethical standards. Written informed consents were provided by all participants or their designees.

**RESEARCH IN CONTEXT**

1. **Systematic review**: Spatially heterogeneous patterns of altered neurometabolism underpin the diverse cognitive impairments seen in Alzheimer's disease (AD). Although local neurometabolic changes are reported in AD through single voxel proton magnetic resonance spectroscopy (^1^H‐MRS) studies, spatial relationships between neurometabolic topography and cognitive impairment remain unexplored.
2. **Interpretation**: Utilizing a novel whole‐brain high‐resolution proton magnetic resonance spectroscopic imaging (^1^H‐MRSI) technique, our study unveiled spatially distinct reductions in N‐acetylaspartate and increases in myo‐inositol in 73 AD (22 prodromal and 51 dementia) patients. N‐acetylaspartate topography correlates with global and specific cognitive scores, supporting neuropathologic findings that link neuronal damage to cognitive performance in AD.
3. **Future directions**: These results highlight the potential of whole‐brain high‐resolution ^1^H‐MRSI as a noninvasive metabolic imaging method that could serve as a surrogate biomarker for tracking AD progression and monitoring treatment efficacy. Longitudinal studies are necessary to establish the correlation between changes in neurometabolites as measured by ^1^H‐MRSI and alterations in cognitive performance.

**TABLE 1 alz14137-tbl-0001:** Demographics.

	CN		aMCI		Mild AD		Moderate AD		
Characteristics	Mean ± SD or n (%)	*n*	Mean ± SD or n (%)	*n*	Mean ± SD or n (%)	*n*	Mean ± SD or n (%)	*n*	*p*‐value
Global CDR score	0	44	0.5	22	1	27	2	24	–
Age (years)	67.36 ± 6.53	44	70.72 ± 7.11	22	69.04 ± 6.51	27	66.25 ± 7.21	24	0.11
Sex (% female)	24 (54.54)	44	14 (63.63)	22	14 (51.85)	27	16 (66.67)	24	0.65
Education (years)	12.39 ± 3.10	44	12.32 ± 3.11	22	11.26 ± 2.84	27	10.54 ± 3.27	24	0.08
Aβ PET global SUVR^*^	0.95 ± 0.05	44	1.41 ± 0.25	22	1.43 ± 0.24	27	1.38 ± 0.21	24	<0.001
Aβ positive/negative	0/44	44	22/0	22	27/0	27	24/0	24	–
MMSE^†^	29.39 ± 0.89	44	26.86 ± 1.55	22	22.11 ± 2.59	27	15.25 ± 4.58	24	<0.001
AVLT immediate recall^‡^	19.86 ± 6.58	21	14.29 ± 3.60	14	7.80 ± 3.74	10	5.50 ± 1.90	10	< 0.001
AVLT total score^§^	32.33 ± 12.22	21	18.79 ± 8.05	14	8.11 ± 5.53	9	5.71 ± 1.50	7	< 0.001
RCFT delayed recall^¶^	20.50 ± 7.47	22	10.15 ± 6.05	13	2.00 ± 2.26	10	2.23 ± 3.22	13	<0.001
RCFT copy^#^	34.14 ± 3.09	22	32.62 ± 7.02	13	23.30 ± 12.95	10	13.77 ± 11.78	13	<0.001
TMT A^**^	4.07 ± 0.47	22	4.26 ± 0.30	14	4.95 ± 0.54	9	5.03 ± 0.26	6	<0.001

Abbreviations: Aβ, amyloid‐β; AD, Alzheimer's disease; aMCI = amnestic mild cognitive impairment; AVLT, Auditory Verbal Learning Test; CDR, Clinical Dementia Rating; CN, cognitively normal; MCI, mild cognitive impairment; MMSE, Mini‐Mental State Examination; PET = positron emission tomography; RCFT, Rey‐Osterrieth Complex Figure Test; SD = standard deviation; SUVR, standard uptake value ratio; TMT A, Trail Making Test Parts A.

*Note*: The CN individuals are amyloid‐β negative, and patients with aMCI, mild AD and moderate AD are amyloid‐β positive. Values are presented as mean ± SD. TMT A scores are shown after log transformation. *P*‐values for age, sex, and education years are obtained from analysis of variance, with post hoc Tukey tests for continuous variables or chi‐squared test for categorical variable. *P*‐values for Aβ PET global SUVR and neuropsychological test scores are derived from analysis of covariance, comparing scores in the aMCI, mild AD, and moderate AD groups with those in the CN group. The analysis of covariance controlled for age, sex, and education years. *P*‐values from post hoc tests are Bonferroni‐corrected for multiple comparisons.

* Aβ+ aMCI < Aβ‐ CN, *p* < 0.001, Aβ+ mild AD < Aβ‐ CN, *p* < 0.001, Aβ+ moderate AD < Aβ‐ CN, *p* < 0.001.

^†^ Aβ+ aMCI < Aβ‐ CN, *p* < 0.001, Aβ+ mild AD < Aβ‐ CN, *p* < 0.001, Aβ+ moderate AD < Aβ‐ CN, *p* < 0.001.

^‡^ Aβ+ mild AD < Aβ‐ CN, *p* < 0.001, Aβ+ moderate AD < Aβ‐ CN, *p* < 0.001.

^§^ Aβ+ aMCI < Aβ‐ CN, *p* < 0.05, Aβ+ mild AD < Aβ‐ CN, *p* < 0.001, Aβ+ moderate AD < Aβ‐ CN, *p* < 0.001.

^¶^ Aβ+ aMCI < Aβ‐ CN, *p* < 0.01, Aβ+ mild AD < Aβ‐ CN, *p* < 0.001, Aβ+ moderate AD < Aβ‐ CN, *p* < 0.001.

^#^ Aβ+ mild AD < Aβ‐ CN, *p* < 0.05, Aβ+ moderate AD < Aβ‐ CN, *p* < 0.001.

** Aβ+ mild AD < Aβ‐ CN, *p* < 0.001, Aβ+ moderate AD < Aβ‐ CN, *p* < 0.001.

### 2.2 Neuropsychological assessments

Participants underwent a neuropsychological test battery that assessed global cognition (MMSE; scored on a scale 0‐30, with 30 indicates least impairment), verbal episodic memory (Auditory Verbal Learning Test [AVLT] immediate recall; scored in total number of words recalled immediately from trial 1‐3. AVLT total score; scored in total number of words from immediate recall, short delayed recall and long‐delayed recall, higher score indicates less impairment),^33^ visual episodic memory (Rey‐Osterrieth Complex Figure Test [RCFT] delayed recall; scores of drawing figures from memory range from 0 to 36, with 36 indicates least impairment), visuospatial function (RCFT copy; scores of drawing figure in the copy trial range from 0 to 36, with 36 indicates least impairment) and attention/processing speed (Trail Making Test Parts A [TMT A]; scored in seconds for the completion time, lower score indicates better performance). The TMT A score was log‐transformed due to non‐normal distribution. Only participants who completed the entire task were included in the subsequent analysis for each neuropsychological test, and the number of participants for each test is listed in Table 1.

### 2.3 Image acquisition

MRI/MRSI and PET images were acquired simultaneously using a 3T Biograph mMR hybrid PET/MR system (Siemens Healthcare, Erlangen, Germany), equipped with a 12‐channel phase‐array head coil. The acquisition scheme of ^18^F‐florbetapir PET and MRI images is shown in Figure S1. As displayed in the figure, right after localizer and MARC Dixon, PET images (acquisition time: 15 min) were acquired together with structural MRI images (acquisition time: 5 min and 4 s) and MRSI images (acquisition time: 9 min and 55 s). Subjects were instructed to lie still with their eyes closed and remain awake during the scan.[Table alz14137-tbl-0001]

3D ^1^H‐MRSI data were acquired using the SPICE sequence with the following parameters: field of view (FOV) = 240.0 × 240.0 × 96.0 mm^3^, voxel size = 2.0 × 3.0 × 3.0 mm^3^, repetition time (TR) = 160 ms, echo time (TE) = 1.60 ms, scan time = 9 min and 55 s. The SPICE technique incorporates several innovative aspects, including: (1) elimination of water and lipid suppression pulses to accelerate imaging; (2) utilization of free induction decay‐based acquisition with ultrashort TE and short TR; (3) adoption of echo‐planar spectroscopic imaging‐based trajectory for rapid spatiotemporal encodings by sampling the (k, t)‐space. In the conducted experiments, full sampling of the central k‐space was implemented to preserve the signal‐to‐noise ratio of metabolite signals, while sparse sampling of the peripheral (k, t)‐space using blipped gradients was employed to achieve high‐resolution for water and lipid signals. The ultrashort TE acquisitions also contributed to an enhancement in signal‐to‐noise ratio. Meanwhile, the combination of short repetition time and sparse sampling of (k, t)‐space facilitated rapid data acquisition. To bolster reliability, a series of navigator signals were acquired at 10‐s intervals to monitor B_0_ field drift and subject head motion. The T1‐weighted structural MR images were acquired with magnetization‐prepared rapid gradient echo (MPRAGE) sequence (FOV = 256.0 × 256.0 × 192.0 mm^3^, voxel size = 0.5 × 0.5 × 1.0 mm^3^, TR = 1900 ms, TE = 2.44 ms, flip angle = 9°).

In the same scan, ^18^F‐florbetapir PET images were acquired, with a matrix size of 344 × 344 × 127 and a voxel size of 2.1 × 2.1 × 2.0 mm^3^. The injected dose of ^18^F‐florbetapir was 238.8 ± 41.1 MBq (range 148.0–366.3 MBq). The PET data were acquired at 45–60 min post a bolus injection. Corrections of random coincidences, dead time, scatter and photon attenuation were applied.^34^ Attenuation correction was implemented using the Dixon method with an additional model‐based bone compartment to improve the precision of standard uptake value (SUV) estimation.^35^ PET images were reconstructed using the ordered subset expectation maximization algorithm and a 2‐mm full‐width half‐maximum Gaussian filter.

### 2.4 Image processing and analysis

The spatiospectral function, containing the relevant neurometabolic information, was reconstructed from the MRSI data collected using the SPICE sequence, leveraging a union‐of‐subspaces model that integrates pre‐learned spectral basis functions.^20^, ^22^, ^23^, ^24^, ^25^, ^26^ Concurrently acquired navigator signals were employed to detect and correct subject head motion and magnetic field drift occurring during the MRSI scan. Corrections for frequency shifts induced by magnetic field inhomogeneity and susceptibility effects were achieved using a high‐resolution field map derived from accompanying water signals. Spectral quantification was performed using an improved LCModel‐based algorithm that incorporates both spatial and spectral priors.^23^ The estimated concentrations of metabolites were normalized with respect to the water reference to compensate for B_1_ inhomogeneity inherent to the head coil utilized in the experiment. Neurometabolites concentrations normalized over the unsuppressed water signal were used for further analysis.

We delineated 15 regions of interest (ROIs) from neocortex and limbic system on MR images, including 10 cortical ROIs (prefrontal cortex, sensorimotor cortex, superior parietal cortex, inferior parietal cortex, precuneus, anterior cingulate cortex, posterior cingulate cortex, superior temporal cortex, middle and inferior temporal cortex, and medial temporal cortex) and five subcortical ROIs (hippocampus, amygdala, thalamus, striatum, and fornix). These regions were defined using the Desikan‐Killiany atlas and subcortical atlas in FreeSurfer (v.6.0),^36^, ^37^ along with the fornix delineated using the JHU atlas. All masks were co‐registered to MRSI maps using ANTs.^38^ The PET images were co‐registered to the structural images with a rigid transformation using ANTs. Standardized uptake value ratio (SUVR) maps were generated through a voxel‐level intensity normalization by taking the ratio to the mean value of the cerebellum. Subsequently, the mean SUVR values were calculated in four cortical regions, that is, frontal cortex, anterior/posterior cingulate cortex, lateral parietal cortex, and lateral temporal cortex. The mean SUVR values in these regions were then averaged to generate a global SUVR.^27^ The participants were stratified into Aβ‐positive or Aβ‐negative using a previously established threshold of 1.11.^28^

To perform voxel‐wise between‐group comparison and voxel‐wise regression analyses, the T1‐weighted MPRAGE images were spatially normalized to the Montreal Neurological Institute (MNI) space, and MRSI maps were co‐registered to MPRAGE images and then normalized to the MNI space using the transform matrix derived from structural image normalization. Both registration processes were implemented using ANTs. The spatially normalized MRSI maps were smoothed by a 10 mm full‐width at half‐maximum Gaussian kernel. For voxel‐wise analysis, the neurometabolic maps and neuropsychological test scores were converted to W‐scores adjusting for age and sex.

### 2.5 Statistical analysis

Demographics were compared between groups using analysis of variance with post hoc Tukey tests for continuous measures and chi‐squared tests for categorical measures. Clinical assessments, neurometabolic levels and SUVR values within each patient group were compared with the CN group using analysis of covariance (ANCOVA), with age, sex and education years as covariates. Shapiro‐Wilk tests were conducted to test data normality, and a nonparametric ANCOVA analysis was employed when the residuals departed from normality. We applied false discovery rate (FDR) corrections using the Benjamini‐Hochberg method to control for multiple testing. Bonferroni corrections were used for post hoc pairwise multiple comparisons. Linear regression was used to assess the correlations of neurometabolic levels and SUVR values with cognitive measures. The cognitive test scores were regressed on regional neurometabolic levels or regional SUVR values, adjusting for age, sex, and education years. The standardized coefficient value of neurometabolic levels for each region was then applied to the corresponding voxels within specific ROIs for visualization. The analysis was repeated with additional adjustments for global SUVR and total gray matter volume (tGMV). All statistical analyses were performed using SPSS 21. The voxel‐wise analyses were conducted using FSL's randomize program with 5000 permutations (https://fsl.fmrib.ox.ac.uk/fsl). For voxel‐wise between‐group comparison, statistically significant clusters were identified by the threshold‐free cluster enhancement method, correcting for multiple comparisons using family‐wise error (FWE) rate with *p* < 0.05. For voxel‐wise regression, the statistically significant clusters were identified using a cluster size threshold corresponding to a false‐positive rate of α  <  0.05 at a voxel‐wise *p* < 0.01. The cluster size threshold was estimated using Monte Carlo simulations (M = 10,000) implemented by AFNI's 3dClustSim (https://afni.nimh.nih.gov).

To assess the predictive ability of neurometabolites across all regions for cognition, we conducted regressions of cognitive responses on neurometabolic levels. Model selection and regional weight estimation were performed using the least absolute shrinkage and selection operator (LASSO). The LASSO method selects important predictors by shrinking the coefficients of covariates that do not contribute additional predictive information to zero, making it suitable for handling highly correlated variables. The penalty parameter was tuned through ten‐fold cross‐validation, and models were subsequently fit on the entire dataset using the cross‐validated penalty parameter. Permutation tests were performed over the mean neurometabolic levels obtained from each region of interest (ROI), which served as the inputs for the LASSO model. The importance of each feature, that is, regional neurometabolic level, was assessed by the drop in the performance metric in the permuted model. A large drop indicates that the feature is important for the model. Here, we repeated 1000 times to derive an averaged drop in *R* ^2^, serving as the measurement of the importance for each feature. Cross‐validated LASSO and permutation tests were implemented in Python's Scikit‐learn package.^39^

## 3 RESULTS

### 3.1 Demographics

**FIGURE 1:**
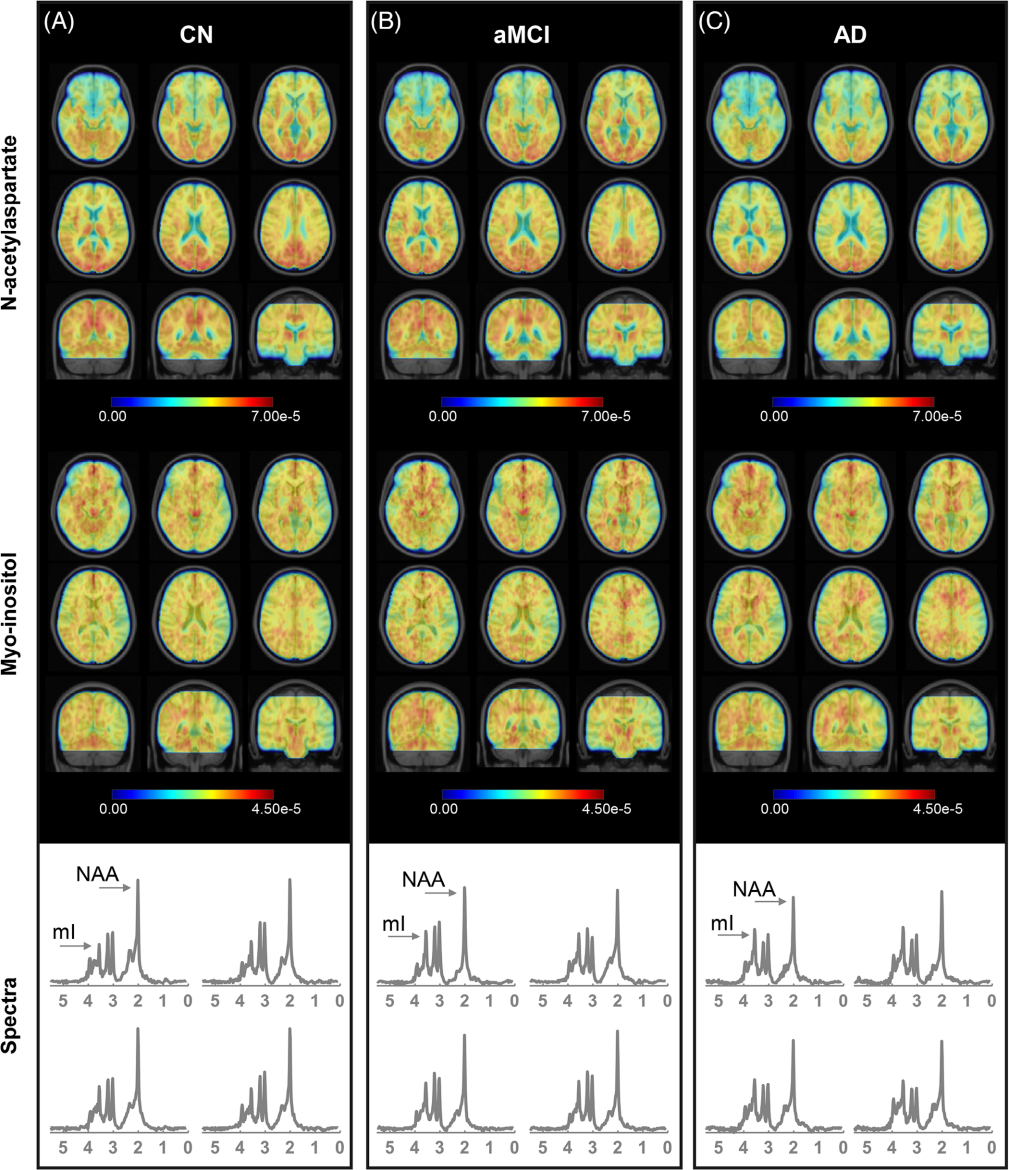
Mean NAA and mI topographies. The mean NAA and mI maps are averaged across (A) CN, (B) aMCI, and (C) AD participants. The maps are superimposed onto the T1‐weighted MNI template. A global reduction in NAA and an elevation in mI are evident in the AD patient group compared with CN individuals. The representative spectra sampled from the precuneus area for a CN subject, an aMCI patient, and an AD patient are presented at the bottom. The AD patient exhibits a notable decrease in the NAA peak and an increase in the mI peak. aMCI, amnestic mild cognitive impairment; AD, Alzheimer's disease; CN, cognitively normal; mI, myo‐inositol; MNI, Montreal Neurological Institute; NAA, N‐acetylaspartate.

One hundred and seventeen participants, including 44 Aβ‐ CN controls, 22 Aβ+ aMCI (i.e., prodromal AD), and 51 Aβ+ AD dementia patients were included (Table 1). The prodromal AD group had a global CDR score of 0.5. Among those diagnosed with AD dementia, 27 manifested mild dementia (CDR = 1), while 24 exhibited moderate dementia (CDR = 2). There were no significant differences in age, sex, or education levels observed between the CN and the prodromal/dementia AD groups. Mean NAA and mI maps of CN, prodromal AD and AD dementia cohorts are presented in Figure 1. The patients with AD dementia exhibited a noticeable global reduction in NAA and elevation in mI compared with the CN subjects.

### 3.2 Comparisons of neurometabolic topography between prodromal AD, AD dementia, and controls

Compared with Aβ‐ CN controls, Aβ+ patients with aMCI or AD exhibited lower NAA levels in the fornix (Cohen's *d* = −0.75, *p* < 0.001), posterior cingulate cortex (Cohen's *d* = −0.64, *p* = 0.006) and precuneus (Cohen's *d* = −0.49, *p* = 0.017). Higher mI levels were observed in the precuneus (Cohen's *d* = 0.73, *p* < 0.001), superior parietal cortex (Cohen's *d* = 0.63, *p* = 0.006), inferior parietal cortex (Cohen's *d* = 0.62, *p* = 0.006), posterior cingulate cortex (Cohen's *d* = 0.55, *p* = 0.010), and middle and inferior temporal cortex (Cohen's *d* = 0.46, *p* = 0.030), as presented in Table 2. The *p*‐values were FDR‐corrected for multiple comparisons.

**TABLE 2:** Regional neurometabolic levels in amyloid‐β positive patients compared with amyloid‐β negative controls.

	NAA				mI			
Regions of interest	Aβ+ patients	Aβ‐ controls	*p*‐value	Cohen's *d* (effect size)	Aβ+ patients	Aβ‐ controls	*p*‐value	Cohen's *d* (effect size)
Prefrontal	0.025 ± 0.002	0.026 ± 0.002	0.068	−0.34	0.020 ± 0.002	0.019 ± 0.002	0.67	0.13
Sensorimotor	0.026 ± 0.002	0.027 ± 0.002	0.16	−0.30	0.018 ± 0.001	0.017 ± 0.001	0.18	0.28
Superior parietal	0.025 ± 0.003	0.026 ± 0.003	0.63	−0.078	0.016 ± 0.002	0.015 ± 0.001	**0.006**	0.63
Inferior parietal	0.024 ± 0.003	0.024 ± 0.002	0.25	−0.23	0.016 ± 0.002	0.015 ± 0.001	**0.006**	0.62
Precuneus	0.027 ± 0.002	0.029 ± 0.002	**0.017**	−0.49	0.019 ± 0.002	0.017 ± 0.002	**<0.001**	0.73
Anterior cingulate	0.030 ± 0.003	0.031 ± 0.002	0.097	−0.33	0.024 ± 0.003	0.024 ± 0.002	0.90	0.017
Posterior cingulate	0.028 ± 0.002	0.029 ± 0.002	**0.006**	−0.64	0.020 ± 0.002	0.019 ± 0.002	**0.010**	0.55
Superior temporal	0.025 ± 0.002	0.026 ± 0.002	0.066	−0.37	0.017 ± 0.001	0.017 ± 0.001	0.67	0.11
Middle and inferior temporal	0.018 ± 0.002	0.018 ± 0.002	0.47	−0.13	0.015 ± 0.002	0.014 ± 0.001	**0.030**	0.46
Medial temporal	0.024 ± 0.003	0.024 ± 0.002	0.90	−0.027	0.021 ± 0.002	0.021 ± 0.002	0.87	0.019
Hippocampus	0.029 ± 0.003	0.030 ± 0.002	0.083	−0.36	0.025 ± 0.002	0.024 ± 0.002	0.24	0.32
Amygdala	0.030 ± 0.003	0.031 ± 0.003	0.16	−0.27	0.025 ± 0.003	0.025 ± 0.002	0.90	0.077
Thalamus	0.029 ± 0.002	0.029 ± 0.002	0.57	−0.088	0.021 ± 0.002	0.021 ± 0.001	0.47	0.18
Striatum	0.026 ± 0.002	0.026 ± 0.002	0.057	−0.40	0.020 ± 0.002	0.019 ± 0.002	0.90	0.018
Fornix	0.026 ± 0.003	0.028 ± 0.003	**<0.001**	−0.75	0.022 ± 0.003	0.022 ± 0.003	0.67	0.15

Notes: Data are presented as mean ± SD. *P*‐values are obtained from analysis of covariance conducted between Aβ+ patients and Aβ‐ controls, with age, sex, and education years as covariates. All *p*‐values are false discovery rate‐corrected for multiple comparisons. Significant *p*‐values are shown in bold.

Abbreviations: Aβ‐, amyloid‐β negative; Aβ+, amyloid‐β positive; mI, myo‐inositol; NAA, N‐acetylaspartate; SD, standard deviation.

**FIGURE 2:**
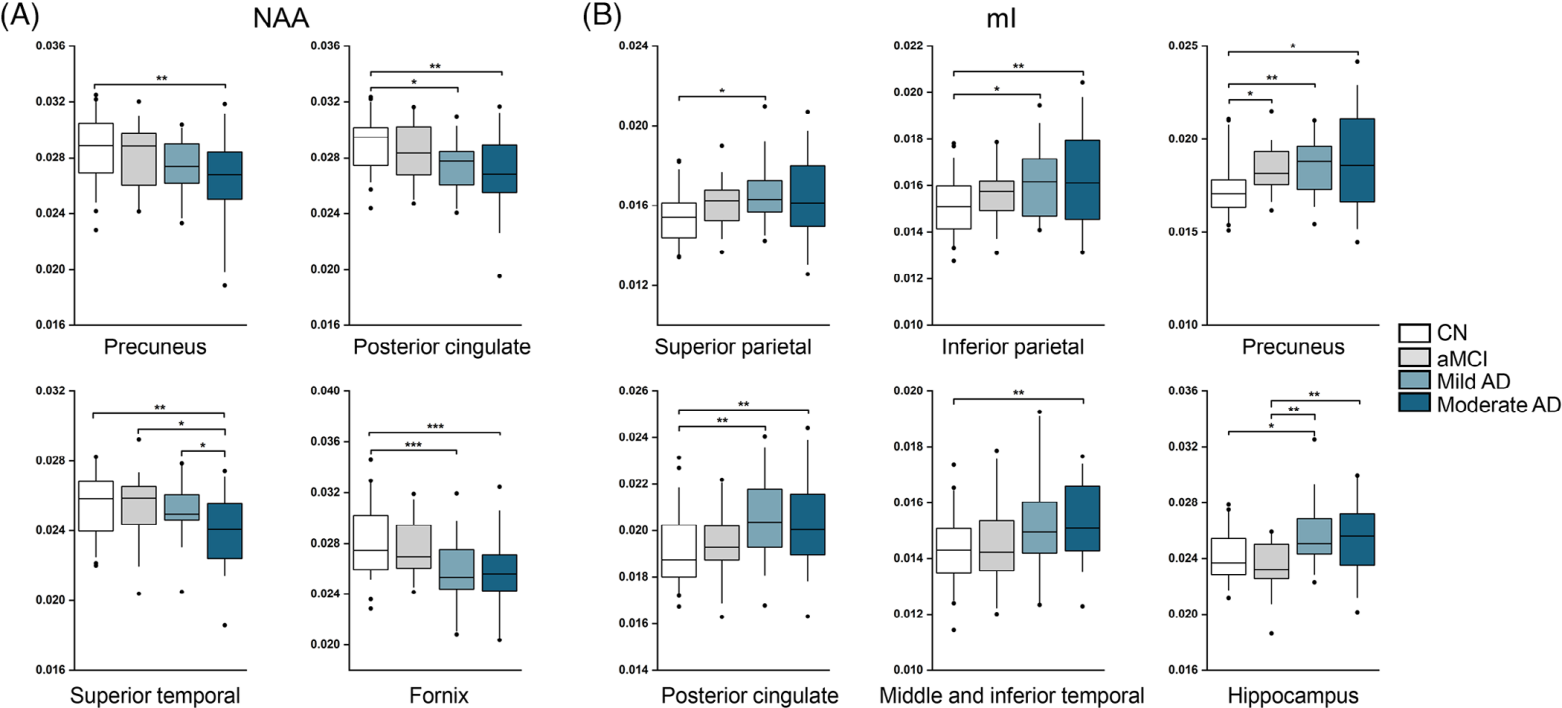
Comparisons of (A) NAA and (B) mI levels between CN, aMCI, mild AD, and moderate AD groups. Boxplots display mean ± interquartile range, with whiskers representing the 5‐95% percentiles. *P*‐values from post hoc pairwise comparisons were Bonferroni‐corrected. *p < 0.05; **p < 0.01; ***p < 0.001. AD, Alzheimer's disease; aMCI, amnestic mild cognitive impairment; CN, cognitively normal; mI, myo‐inositol; NAA, N‐acetylaspartate.

Group comparisons among CN, aMCI, mild AD, and moderate AD groups assessed neurometabolic changes across varying levels of disease severity (Figure 2). NAA was significantly reduced in the moderate AD group in the fornix (*p* < 0.001), superior temporal cortex (*p* = 0.001), precuneus (*p* = 0.004) and posterior cingulate cortex (*p* = 0.005) compared with the CN group. In the mild AD group, NAA reduction was observed in the fornix (*p* < 0.001) and posterior cingulate cortex (*p* = 0.012) compared with the CN group. Comparing the moderate AD group with both mild AD (*p* = 0.038) and aMCI (*p* = 0.050) groups revealed a significant reduction in NAA levels in the superior temporal cortex. The *p*‐values from post hoc tests were Bonferroni‐corrected for multiple comparisons.

Increased mI levels were shown in the precuneus (*p* = 0.033) in the aMCI group compared with CN. In mild AD, elevated mI levels were found in precuneus (*p* = 0.003), posterior cingulate cortex (*p* = 0.003), superior parietal cortex (*p* = 0.013), inferior parietal cortex (*p* = 0.021), and hippocampus (*p* = 0.041). In moderate AD, elevated mI levels were observed in the middle and inferior temporal cortex (*p* = 0.003), inferior parietal cortex (*p* = 0.006), posterior cingulate cortex (*p* = 0.008), and precuneus (*p* = 0.011), in comparison to the CN group. The hippocampus exhibited significantly elevated mI levels in both mild (*p* = 0.003) and moderate AD (*p* = 0.007) groups compared with the aMCI group. The *p*‐values from post hoc tests were Bonferroni‐corrected for multiple comparisons. We did not find significant differences in choline (Cho) and creatine (Cr) levels between patient and control groups after FDR corrections (Table S1). For comparison, we also analyzed the changes in Aβ SUVR values, as shown in Figure S2. There is a significant elevation in Aβ SUVR across all cortical and subcortical areas (excluding the hippocampus) in patient cohorts compared to the control group. No significant differences in SUVR were observed between any two patient cohorts.

The results of voxel‐wise comparisons of NAA and mI are shown in Figure S3. In addition to the regions identified in the region‐wise analysis, reduced NAA levels were also observed in the inferior frontal gyri, middle temporal gyri, and inferior parietal lobule. Increased mI levels were observed in the regions reported in the region‐wise analysis, as well as in the superior temporal gyri and thalamus.

### 3.3 Associations between neurometabolic topography and cognitive functions

Regional correlations between neurometabolic levels and cognitive measures in Aβ+ patients are presented in Table 3. The MMSE score exhibited a positive relationship with NAA in a broad area, encompassing the superior temporal cortex (B = 0.33, *p* = 0.003), thalamus (B = 0.32, *p* = 0.005), precuneus (B = 0.29, *p* = 0.014), posterior cingulate cortex (B = 0.26, *p* = 0.022), fornix (B = 0.26, *p* = 0.022), inferior parietal cortex (B = 0.26, *p* = 0.028), sensorimotor cortex (B = 0.24, *p* = 0.033), anterior cingulate cortex (B = 0.24, *p* = 0.035), and hippocampus (B = 0.24, *p* = 0.037). Additionally, MMSE demonstrated a negative relationship with mI in the inferior parietal cortex (B = −0.24, *p* = 0.031), middle and inferior temporal cortex (B = −0.25, *p* = 0.038), and hippocampus (B = −0.23, *p* = 0.036). All associations remained significant after adjusting for global SUVR, and the associations for NAA levels in the thalamus, hippocampus, superior temporal cortex, and fornix remained significant after adjusting for tGMV or for both global SUVR and tGMV. The results of voxel‐wise correlations of NAA and mI with MMSE are shown in Figure S4A. In addition to the regions identified in the region‐wise analysis, reduced NAA levels were also associated with MMSE in the middle temporal gyrus. Increased mI levels were associated with MMSE in the superior temporal gyrus and parahippocampus, extending beyond the regions reported in the region‐wise analysis.

**TABLE 3:** Associations between regional neurometabolic levels and cognitive measures in prodromal AD and AD dementia patients

	MMSE			AVLT immediate recall			AVLT total score			RCFT delayed recall			RCFT copy			TMT A		
NAA	B	95% CI	*p*‐value	B	95% CI	*p*‐value	B	95% CI	*p*‐value	B	95% CI	*p*‐value	B	95% CI	*p*‐value	B	95% CI	*p*‐value
Prefrontal	0.14	[−0.10, 0.37]	0.25	0.46	[−0.02, 0.93]	0.058	0.41	[−0.15, 0.98]	0.15	0.18	[−0.23, 0.59]	0.38	0.32	[−0.08, 0.72]	0.11	−0.36	[−0.86, 0.13]	0.14
Sensorimotor	0.24	[0.02, 0.46]	**0.033** ^*^	0.48	[0.07, 0.88]	**0.022** ^*^, ^†^, ^‡^	0.40	[−0.11, 0.91]	0.12	0.35	[−0.01, 0.70]	0.053^*^	0.36	[0.01, 0.72]	**0.046** ^†^	−0.27	[−0.75, 0.21]	0.26
Superior parietal	0.21	[−0.01, 0.43]	0.065	0.37	[−0.03, 0.77]	0.065	0.28	[−0.18, 0.74]	0.22	0.34	[0.003, 0.68]	**0.048** ^*^	0.39	[0.05, 0.73]	**0.026** ^*^	−0.41	[−0.82, 0]	0.051
Inferior parietal	0.26	[0.03, 0.49]	**0.028** ^*^	0.52	[0.12, 0.92]	**0.012** ^*^, ^†^, ^‡^	0.47	[−0.01, 0.95]	0.052	0.53	[0.21, 0.86]	**0.002** ^*^, ^†^, ^‡^	0.35	[−0.01, 0.71]	0.058^†^	−0.33	[−0.78, 0.12]	0.15
Precuneus	0.29	[0.06, 0.52]	**0.014** ^*^	0.44	[0.05, 0.83]	**0.027** ^*^	0.34	[−0.12, 0.79]	0.14	0.27	[−0.08, 0.62]	0.12	0.34	[−0.01, 0.68]	0.056	−0.58	[−1.01, −0.15]	**0.011** ^*^
Anterior cingulate	0.24	[0.02, 0.47]	**0.035** ^*^	0.29	[−0.09, 0.66]	0.13	0.26	[−0.15, 0.68]	0.20	0.11	[−0.22, 0.45]	0.50	0.17	[−0.17, 0.51]	0.31	−0.02	[−0.43, 0.39]	0.93
Posterior cingulate	0.26	[0.04, 0.49]	**0.022** ^*^	0.50	[0.10, 0.91]	**0.017** ^*^, ^†^	0.30	[−0.22, 0.82]	0.24	0.32	[−0.05, 0.69]	0.090	0.44	[0.08, 0.80]	**0.018** ^*^	−0.56	[−1.05, −0.07]	**0.026** ^*^
Superior temporal	0.33	[0.11, 0.55]	**0.003** ^*^, ^†^, ^‡^	0.50	[0.11, 0.89]	**0.014** ^*^, ^†^, ^‡^	0.43	[−0.01, 0.88]	0.057	0.37	[0.02, 0.71]	**0.038** ^*^	0.31	[−0.05, 0.67]	0.087	−0.22	[−0.67, 0.23]	0.32
Middle and inferior temporal	0.09	[−0.15, 0.33]	0.45	−0.02	[−0.47, 0.43]	0.92	−0.11	[−0.61, 0.39]	0.66	0.24	[−0.13, 0.60]	0.20	0.02	[−0.36, 0.40]	0.90	−0.03	[−0.52, 0.47]	0.91
Medial temporal	−0.07	[−0.30, 0.16]	0.55	−0.06	[−0.45, 0.33]	0.77	−0.07	[−0.50, 0.37]	0.75	−0.01	[−0.35, 0.32]	0.93	−0.13	[−0.46, 0.21]	0.45	−0.03	[−0.47, 0.42]	0.91
Hippocampus	0.24	[0.02, 0.46]	**0.037** ^*^, ^†^, ^‡^	0.55	[0.23, 0.88]	**0.002** ^*^, ^†^, ^‡^	0.46	[0.06, 0.85]	**0.026** ^*^, ^†^, ^‡^	0.20	[−0.14, 0.53]	0.24	0.26	[−0.07, 0.59]	0.12	−0.37	[−0.80, 0.07]	0.10
Amygdala	0.16	[−0.07, 0.39]	0.18	0.59	[0.26, 0.92]	**0.001** ^*^, ^†^, ^‡^	0.53	[0.16, 0.91]	**0.007** ^*^, ^†^, ^‡^	0.15	[−0.19, 0.50]	0.36	0.24	[−0.10, 0.57]	0.16	−0.33	[−0.75, 0.08]	0.11
Thalamus	0.32	[0.10, 0.54]	**0.005** ^*^, ^†^, ^‡^	0.54	[0.21, 0.88]	**0.003** ^*^, ^†^, ^‡^	0.56	[0.17, 0.95]	**0.007** ^*^, ^†^, ^‡^	0.28	[−0.04, 0.61]	0.086	0.29	[−0.03, 0.62]	0.075	−0.33	[−0.77, 0.12]	0.14
Striatum	0.21	[−0.02, 0.43]	0.068	0.48	[0.15, 0.81]	**0.006** ^*^, ^†^, ^‡^	0.43	[0.05, 0.80]	**0.027** ^*^, ^†^, ^‡^	0.19	[−0.14, 0.51]	0.25	0.16	[−0.16, 0.49]	0.31	−0.30	[−0.71, 0.10]	0.14
Fornix	0.26	[0.04, 0.48]	**0.022** ^*^, ^†^, ^‡^	0.54	[0.18, 0.90]	**0.004** ^*^, ^†^, ^‡^	0.44	[0.02, 0.87]	**0.041** ^*^	0.31	[−0.03, 0.65]	0.072	0.27	[−0.08, 0.62]	0.13	−0.76	[−1.12, −0.41]	**<0.001** ^*^, ^†^, ^‡^

	MMSE			AVLT immediate recall			AVLT total score			RCFT delayed recall			RCFT copy			TMT A		
mI	B	95% CI	*p*‐value	B	95% CI	*p*‐value	B	95% CI	*p*‐value	B	95% CI	*p*‐value	B	95% CI	*p*‐value	B	95% CI	*p*‐value
Prefrontal	−0.21	[−0.43, 0.01]	0.063	−0.07	[−0.47, 0.34]	0.74	−0.17	[−0.65, 0.31]	0.48	−0.21	[−0.55, 0.14]	0.24	−0.17	[−0.52, 0.19]	0.35	0.16	[−0.29, 0.62]	0.46
Sensorimotor	−0.09	[−0.32, 0.14]	0.42	0.02	[−0.41, 0.45]	0.94	−0.09	[−0.60, 0.41]	0.71	0.22	[−0.15, 0.59]	0.24	0.09	[−0.29, 0.47]	0.62	−0.13	[−0.60, 0.34]	0.59
Superior parietal	−0.12	[−0.34, 0.11]	0.30	0.16	[−0.25, 0.56]	0.44	0.09	[−0.38, 0.55]	0.71	0.16	[−0.19, 0.51]	0.35	0.06	[−0.29, 0.42]	0.72	−0.14	[−0.59, 0.31]	0.53
Inferior parietal	−0.24	[−0.46, −0.02]	**0.031** ^*^	0.18	[−0.22, 0.58]	0.37	0.11	[−0.34, 0.57]	0.61	0.13	[−0.22, 0.47]	0.45	−0.04	[−0.39, 0.31]	0.82	0.12	[−0.33, 0.56]	0.60
Precuneus	−0.15	[−0.37, 0.08]	0.19	0.11	[−0.31, 0.54]	0.59	0.01	[−0.49, 0.51]	0.96	0.03	[−0.33, 0.39]	0.87	−0.08	[−0.44, 0.28]	0.66	0.36	[−0.11, 0.83]	0.13
Anterior cingulate	0.006	[−0.22, 0.23]	0.96	0.09	[−0.32, 0.49]	0.67	0.02	[−0.43, 0.47]	0.94	−0.11	[−0.45, 0.24]	0.54	−0.10	[−0.45, 0.25]	0.57	0.06	[−0.37, 0.49]	0.77
Posterior cingulate	−0.16	[−0.39, 0.06]	0.16	−0.14	[−0.54, 0.27]	0.49	−0.23	[−0.68, 0.22]	0.30	0.03	[−0.33, 0.38]	0.87	0.01	[−0.35, 0.37]	0.96	0.29	[−0.14, 0.72]	0.18
Superior temporal	−0.12	[−0.35, 0.11]	0.30	0.10	[−0.29, 0.50]	0.60	0.03	[−0.43, 0.50]	0.89	−0.13	[−0.48, 0.23]	0.47	−0.15	[−0.51, 0.20]	0.39	0.20	[−0.25, 0.65]	0.37
Middle and inferior temporal	−0.25	[−0.48, −0.01]	**0.038** ^*^	−0.31	[−0.70, 0.09]	0.12	−0.34	[−0.79, 0.10]	0.13	−0.23	[−0.59, 0.14]	0.22	−0.38	[−0.73, −0.03]	**0.034** ^*^	0.42	[−0.07, 0.91]	0.088
Medial temporal	−0.23	[−0.46, 0]	0.05	−0.14	[−0.53, 0.26]	0.48	−0.13	[−0.57, 0.32]	0.57	−0.25	[−0.58, 0.09]	0.14	−0.26	[−0.60, 0.08]	0.13^‡^	0.14	[−0.32, 0.61]	0.53
Hippocampus	−0.23	[−0.45, −0.02]	**0.036** ^*^	−0.14	[−0.52, 0.23]	0.45	−0.24	[−0.64, 0.16]	0.23	−0.25	[−0.57, 0.07]	0.12	−0.04	[−0.38, 0.30]	0.80	0.24	[−0.16, 0.64]	0.23
Amygdala	−0.17	[−0.39, 0.05]	0.14	0.21	[−0.18, 0.59]	0.28	0.17	[−0.26, 0.59]	0.43	−0.14	[−0.48, 0.20]	0.41	−0.07	[−0.42, 0.29]	0.71	−0.15	[−0.57, 0.27]	0.48
Thalamus	−0.07	[−0.31, 0.16]	0.54	−0.12	[−0.51, 0.27]	0.54	−0.18	[−0.62, 0.26]	0.41	−0.12	[−0.46, 0.23]	0.49	0.04	[−0.31, 0.39]	0.81	0.20	[−0.21, 0.61]	0.33
Striatum	−0.12	[−0.35, 0.12]	0.32	−0.01	[−0.41, 0.39]	0.96	−0.14	[−0.61, 0.33]	0.55	−0.24	[−0.58, 0.10]	0.16	−0.03	[−0.38, 0.33]	0.88	0.04	[−0.42, 0.50]	0.86
Fornix	−0.05	[−0.28, 0.18]	0.67	0.07	[−0.30, 0.45]	0.69	−0.12	[−0.53, 0.29]	0.55	−0.13	[−0.46, 0.20]	0.44	0.1	[−0.24, 0.43]	0.56	−0.15	[−0.56, 0.26]	0.45

*Note*: Standardized coefficients (B), 95% confidence intervals (95% CI), and *p*‐values derived from linear regression models are reported. In the linear regression model, cognitive measure was regressed on the regional neurometabolic level, adjusting for age, sex, and education years. Significant *p*‐values are in bold.

Abbreviations: AD, Alzheimer's disease; AVLT, Auditory Verbal Learning Test; CI = confidence interval; mI, myo‐inositol; MMSE, Mini‐Mental State Examination; NAA, N‐acetylaspartate; RCFT, Rey‐Osterrieth Complex Figure Test; SUVR = standardized uptake value ratio; TMT A, Trail Making Test Parts A.

* *P* < 0.05 when also adjusting the models for global SUVR.

^†^ *P* < 0.05 when also adjusting the models for total gray matter volume.

^‡^ *P* < 0.05 when also adjusting the models for both global SUVR and total gray matter volume.

The associations with specific neuropsychological measures were predominantly linked to NAA levels. The AVLT immediate recall score was significantly associated with NAA levels in the amygdala (B = 0.59, *p* = 0.001), hippocampus (B = 0.55, *p* = 0.002), thalamus (B = 0.54, *p* = 0.003), fornix (B = 0.54, *p* = 0.004), inferior parietal cortex (B = 0.52, *p* = 0.012), superior temporal cortex (B = 0.50, *p* = 0.014), posterior cingulate cortex (B = 0.50, *p* = 0.017), striatum (B = 0.48, *p* = 0.006), sensorimotor cortex (B = 0.48, *p* = 0.022), and precuneus (B = 0.44, *p* = 0.027). NAA exhibited an association with AVLT total score in the thalamus (B = 0.56, *p* = 0.007), amygdala (B = 0.53, *p* = 0.007), hippocampus (B = 0.46, *p* = 0.026), fornix (B = 0.44, *p* = 0.041), and striatum (B = 0.43, *p* = 0.027). Regarding RCFT delayed recall, a significant association was observed for NAA levels in the inferior parietal cortex (B = 0.53, *p* = 0.002), superior temporal cortex (B = 0.37, *p* = 0.038), and superior parietal cortex (B = 0.34, *p* = 0.048). Lower RCFT copy score was associated with lower NAA in the posterior cingulate cortex (B = 0.44, *p* = 0.018), superior parietal cortex (B = 0.39, *p* = 0.026), and sensorimotor cortex (B = 0.36, *p* = 0.046), and was associated with higher mI in the middle and inferior temporal cortex (B = −0.38, *p* = 0.034). Notably, regions significantly associated with TMT A score were the fornix (B = −0.76, *p* < 0.001), precuneus (B = −0.58, *p* = 0.011), and posterior cingulate cortex (B = −0.56, *p* = 0.026) for NAA.

**FIGURE 3:**
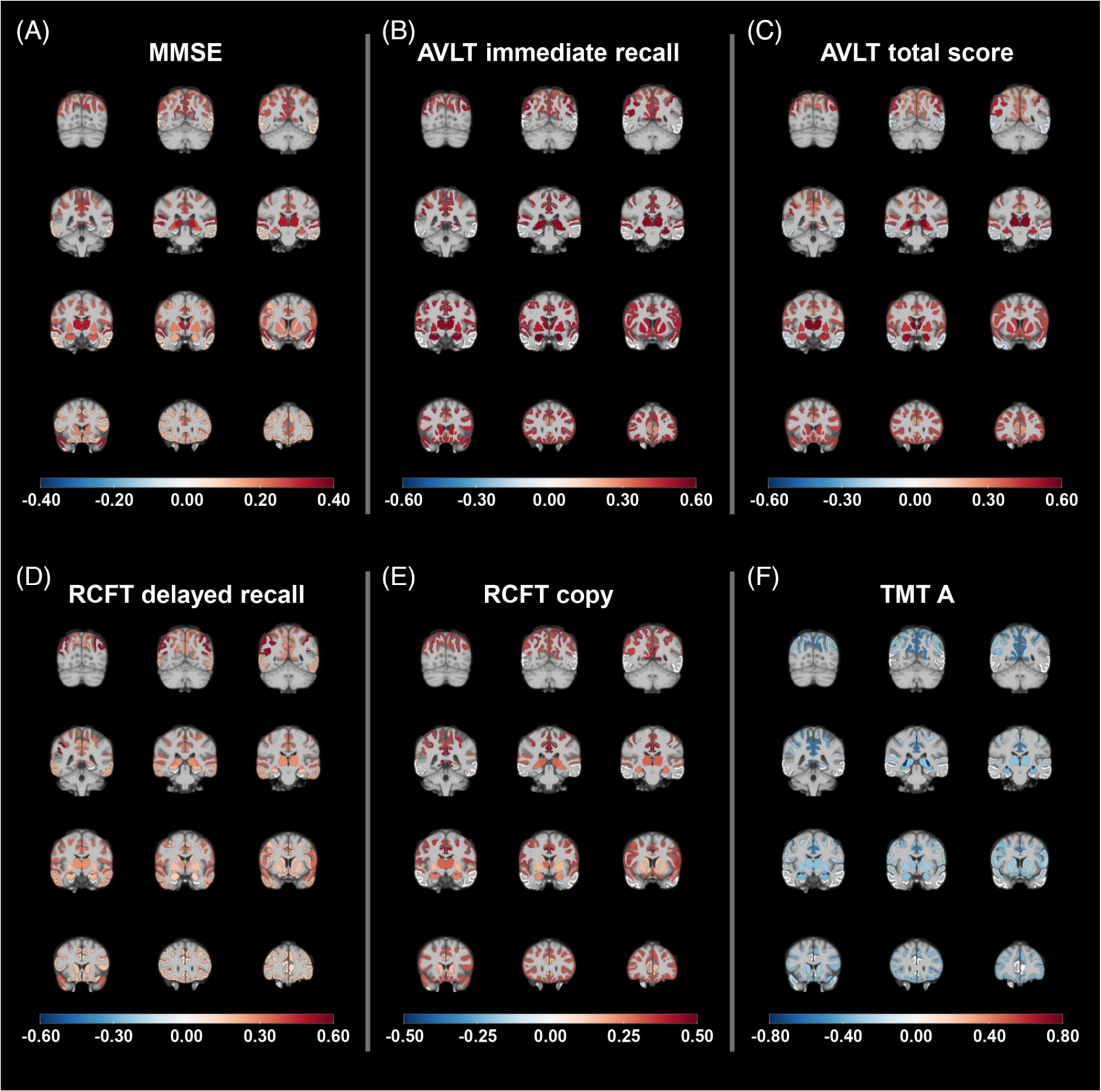
Association maps of NAA and cognitive measures in all the regions of interest for prodromal AD (amyloid‐β positive aMCI) and AD dementia patients. Standardized coefficient from linear regression model was calculated for the association between NAA level and (A) global cognition (MMSE), as well as domain specific cognitive performance including (B) AVLT immediate recall, (C) AVLT total score, (D) RCFT delayed recall, (E) RCFT copy, and (F) TMT A scores. Cognitive measures were regressed on regional NAA levels, adjusting for age, sex, and education years. Brain maps depict region‐specific association outcomes, generated by assigning a uniform color to each voxel within specified regions of interest based on the calculated standardized coefficient for the entire region. These maps were overlaid onto a T1‐weighted MNI template. AD, Alzheimer's disease; aMCI, amnestic mild cognitive impairment; AVLT, Auditory Verbal Learning Test; MMSE, Mini‐Mental State Examination; MNI, Montreal Neurological Institute; NAA, N‐acetylaspartate; RCFT, Rey‐Osterrieth Complex Figure Test; TMT A, Trail Making Test Parts A.

Regional patterns of association between NAA and each cognitive measure were presented in Figure 3. A uniform color was assigned to each voxel within specified ROIs based on the calculated standardized coefficient for the entire region. The associations remained significant after adjusting for global SUVR, with the exception of sensorimotor cortex in the RCFT copy test. AVLT immediate recall and total scores retained significance after adjusting for either tGMV or both global SUVR and tGMV, except in the precuneus and posterior cingulate cortex for AVLT immediate recall and the fornix for AVLT total score. The inferior parietal cortex for RCFT delayed recall and the fornix for TMT A maintained significant correlations when adjusting for tGMV or both global SUVR and tGMV.

As a comparison, we also analyzed the association between SUVR and cognitive functions, presented in Table S2. A negative association was found between hippocampus SUVR value and TMT A (B = −0.39, *p* = 0.046). After adjusting for total gray matter volume, the hippocampus SUVR value was associated with MMSE (B = 0.22, *p* = 0.027). The weak correlations between Aβ SUVR values and cognitive functions are consistent with previous literature.^40^

The results of voxel‐wise correlations of NAA with specific neuropsychological measures are shown in Figure S4B. The regions with significant associations generally align with the findings reported in the region‐wise analysis. Expanding on these findings, NAA levels were associated with AVLT immediate recall in the parahippocampus. Significant clusters related to the AVLT total score were observed in the inferior parietal lobule. RCFT delayed recall and RCFT copy test correlated with NAA in the precuneus, while the RCFT copy test was also associated with NAA in the inferior frontal gyri. The TMT A test score was related to NAA in the superior and middle temporal gyri, as well as the inferior parietal lobule.

### 3.4 Predicting cognitive function from neurometabolic topography

**FIGURE 4:**
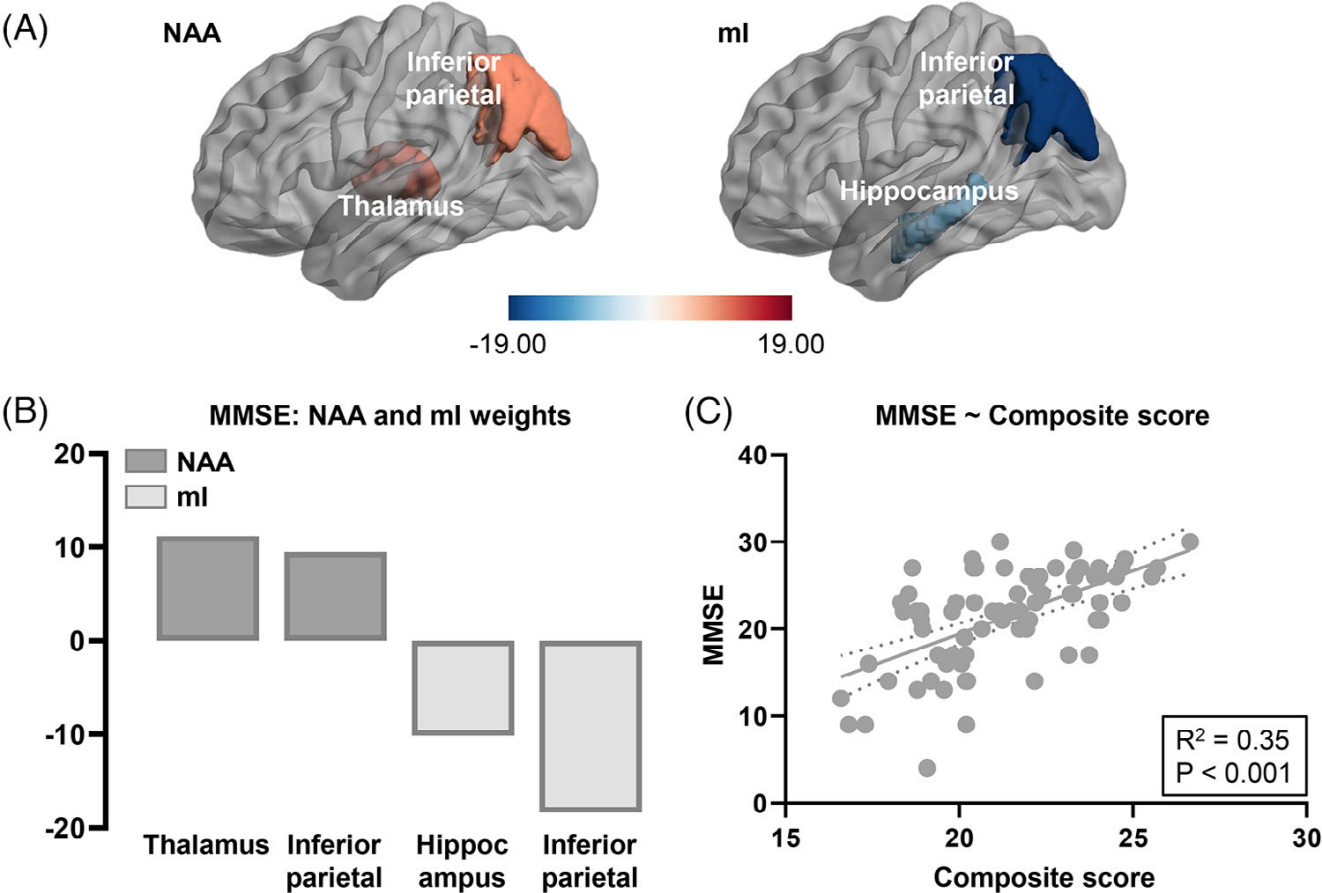
Neurometabolic topography and global cognition in prodromal AD (amyloid‐β positive aMCI) and AD dementia patients. (A) The 3D brain rendering provides a visualization of the model‐selected regions, with color‐coding based on the respective model‐estimated weights for predicting the MMSE score. (B) The model‐estimated regional weights in barplots for NAA and mI. (C) Scatter plot of MMSE score against the composite score fitted from the estimated model. Linear regression curve and 95% confidence interval are shown in the scatter plot. A threshold of 0.02 for permutation feature importance was applied to identify the most important regions from the estimated model. AD, Alzheimer's disease; aMCI, amnestic mild cognitive impairment; mI, myo‐inositol; MMSE, Mini‐Mental State Examination; NAA, N‐acetylaspartate.

**FIGURE 5:**
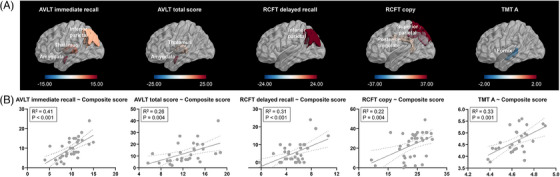
Neurometabolic topography and domain specific cognitive measures in prodromal AD (amyloid‐β positive aMCI) and AD dementia patients. (A) The 3D brain rendering provides a visualization of the model‐selected regions, with color‐coding based on the respective model‐estimated weights for predicting different cognitive scores. A threshold of 0.02 for permutation feature importance was applied to identify the most critical regions from the estimated model. (B) Scatter plots of the cognitive scores against the composite scores fitted from the estimated model. Linear regression curves and 95% confidence intervals are shown in the scatter plot. AD, Alzheimer's disease; aMCI, amnestic mild cognitive impairment; AVLT, Auditory Verbal Learning Test; NAA, N‐acetylaspartate; RCFT, Rey‐Osterrieth Complex Figure Test; TMT A, Trail Making Test Parts A.

Using the LASSO model, we regressed the cognitive measures of AD (prodromal AD and AD dementia) patients on NAA and mI levels across all the ROIs. Combined NAA and mI topographies exhibited significant predictive performance for MMSE (*R* ^2^ = 0.35, *p* < 0.001). The main regions selected included the thalamus and inferior parietal cortex for NAA, and the inferior parietal cortex and hippocampus for mI (Figure 4). The integrated NAA topographies that best predicted each cognitive function are shown in Figure 5. The model showed significant predictive performance for AVLT immediate recall score (*R* ^2^ = 0.41, *p* < 0.001), AVLT total score (*R* ^2^ = 0.26, *p* = 0.004), RCFT delayed recall (*R* ^2^ = 0.31, *p* < 0.001), RCFT copy (*R* ^2^ = 0.22, *p* = 0.004), and TMT A (*R* ^2^ = 0.33, *p* = 0.001). For AVLT immediate recall score, the amygdala, thalamus and inferior parietal cortex were selected as predictive regions. For AVLT total score, the amygdala and thalamus were selected. For RCFT delayed recall, inferior parietal cortex served as the predictive region. For RCFT copy, predictive regions included the superior parietal cortex and posterior cingulate cortex. Additionally, the fornix was identified for TMT A.

## 4 DISCUSSION

In this study, we investigated the spatial distributions of neurometabolites, focusing on NAA and mI, and their associations with cognitive impairments in prodromal AD and AD dementia patients. Our findings reveal spatially heterogeneous reductions in NAA and elevated mI levels in these patients, with more pronounced distinctions in the posterior cingulate and fornix regions, and subtler differences in the prefrontal and superior temporal cortex for NAA levels. Differences in mI were more prominent in the precuneus and parietal cortex, and subtler in the middle and inferior temporal cortex. Reduced NAA and increased mI were associated with worse global cognitive performance, while NAA also exhibited associations with specific cognitive domains (verbal episodic memory, visual episodic memory, attention/processing speed, and visuospatial abilities), particularly in the limbic and temporo‐parietal cortical regions. The neurometabolic topography significantly predicted both global and domain‐specific cognitive performance in prodromal AD and AD dementia patients.

### 4.1 Spatial distributions of neurometabolic abnormality in prodromal AD and AD dementia patients

Our work demonstrates decreased NAA levels and increased mI levels, aligning with previous studies highlighting specific regions such as the precuneus, posterior cingulate cortex, and parietal cortex.^4^, ^41^, ^42^ Additionally, we observed a notable reduction in NAA in the fornix during the early stages of mild AD, progressing to the superior temporal cortex in moderate AD. NAA, an amino acid synthesized primarily in neuronal mitochondria, serves as a marker for neuroaxonal viability, function, and density. Our observed patterns of NAA reduction align with Braak's model of AD pathophysiology, indicating initiation of neuronal damage/dysfunction in the limbic system before spreading to higher cortical regions.

We observed increased mI level in the precuneus of individuals with Aβ+ aMCI compared with CNs, indicating this region as the earliest to manifest changes. In the Aβ+ AD group, mI levels also increased in the posterior cingulate cortex. Previous studies often combined the precuneus and posterior cingulate cortex due to challenges in differentiating these regions with a single voxel. However, our 3D‐MRSI technology's high‐resolution imaging effectively distinguishes neurometabolic changes between these regions. Increased mI levels are biomarkers for glial cell proliferation, with histopathological studies confirming proliferated glial cells near Aβ plaques.^43^, ^44^ Elevated mI levels were also observed in the superior and inferior parietal cortices, as well as the middle and inferior temporal cortex, consistent with findings reported in the temporo‐parietal or parietal regions of AD patients.^5^, ^11^, ^45^, ^46^ Notably, our study found that mI increases in the hippocampus showed a progressive pattern from Aβ+ aMCI to mild AD, consistent with prior observations of Aβ deposition.^47^, ^48^, ^49^

Our study did not observe significant changes in Cho or Cr levels in patients with prodromal AD or AD dementia compared to controls. The results agree with the literature that the clinical relevance of these two biomarkers in AD diagnosis is unclear. Studies have reported conflicting findings about alteration in Cho,^4^, ^50^, ^51^ and Cr is commonly utilized as an internal reference for normalization in MRS studies to account for technical variations.^52^

### 4.2 Associations between regional neurometabolic changes and global cognitive impairment

NAA levels positively correlated with MMSE scores, particularly in parietal‐temporal regions (posterior cingulate cortex, precuneus, inferior parietal cortex, superior temporal cortex, thalamus, and fornix). Levels of mI showed a negative correlation with MMSE, notably in the inferior parietal cortex, hippocampus, and middle and inferior temporal cortex. Previous studies also linked NAA and mI levels in the posterior cingulate gyrus or parietal cortex with MMSE scores in AD patients, indicating progressive neuronal decline or gliosis with dementia severity. Discrepancies in the correlation between NAA in the hippocampus and MMSE may reflect limited sample sizes in early studies, attributable to technical challenges encountered in hippocampal MRS.^53^, ^54^ Patterns of NAA and mI associations align with tau and FDG PET imaging findings. Tau‐PET imaging showed MMSE correlations in superior temporal, parietal, posterior cingulate cortex, and inferior temporal cortex in AD patients.^55^ Similarly, glucose metabolism decline, reflecting synaptic dysfunction and loss, correlated with MMSE scores and was marked by hypometabolism in the precuneus, posterior cingulate, inferior parietal lobule, and medial and inferior temporal gyrus.^56^ Our study supports that in vivo markers of neuronal density/function decrease and gliosis, which are histopathological features of AD, correlate with cognitive impairment, following spatial patterns seen in tau distribution and hypometabolism.

### 4.3 Associations between regional neurometabolic changes and specific cognitive impairments

Reduced NAA levels closely correlate with specific domain cognitive impairments in prodromal AD and AD dementia patients. Verbal episodic memory is linked to NAA reductions in limbic structures such as the hippocampus, fornix, amygdala, thalamus, and striatum, as well as the parietal‐temporal cortex for immediate recall. This aligns with the importance of the Papez circuit, composed of hippocampus, fornix, mammillary bodies, anterior thalamic nuclei, and posterior cingulate, in episodic memory.^57^, ^58^, ^59^, ^60^ Combined MRI and PET imaging show reduced metabolism in these limbic structures in AD patients.^61^ PET studies also highlight activation in temporal and parietal regions during verbal episodic memory retrieval.^62^ Visual episodic memory performance is associated with NAA reductions in the inferior parietal, superior parietal, and superior temporal cortex. Tau PET imaging studies find negative associations between visual memory scores and tau deposition in temporo‐parietal regions.^63^ While the exact role of the nervous system in information storage and retrieval remains incompletely understood, the temporal and parietal lobes are recognized as essential components of the memory network, with increased activity during information recall.^64^ Notably, the parietal lobe, crucial for visuospatial perception, likely plays a key role in the storage of visuospatial information.^65^ Studies in nonhuman primates have illustrated connections between the inferior parietal lobe and both the entorhinal cortex^66^, ^67^ and the hippocampus,^68^ indicating the involvement of the inferior parietal lobe in the memory circuitry.^69^ Our findings support the notion that impaired neuronal metabolism in the temporo‐parietal regions contributes to verbal and visual episodic memory impairment in patients with AD.

Our study found significant correlations between reduced NAA in the fornix and impairments in episodic memory and attention/processing speed. The fornix, a key component of the limbic system, connects the hippocampus to other brain regions. Its small volume (1‐1.8 mL)^70^ makes traditional MRSI challenging, but high‐resolution SPICE imaging (18 μL resolution) revealed changes in NAA levels in the fornix. The fornix is crucial for encoding and recalling diverse memory types and is linked to processing speed.^71^ Disruption of fornix integrity correlates with memory and attention dysfunction in Apolipoprotein E4 carriers.^72^ Clinical trials suggest fornix deep brain stimulation may improve cognitive function in AD patients, highlighting its potential as a neurosurgical target for treating cognitive impairment in AD.^73^, ^74^ Our findings on neuronal loss or dysfunction in the limbic network may provide potential novel targets for AD diagnosis and treatment.

### 4.4 Limitations

Our study has several limitations. First, the diagnosis of AD relied on standard clinical criteria combined with amyloid PET positivity, without assessing biomarkers of tau pathogenesis or neuropathology at autopsy. The lack of tau measurement precluded an exploration of the impact of tau pathology on neurometabolites and their associations with cognition. Due to the restricted accessibility of tau‐PET imaging, the subjects in our study were not stratified into tau positive or negative. Tau‐mediated neurofibrillary tangle distribution serves as the defining characteristics of Braak staging, which is closely related to clinical symptoms. Therefore, future investigations should consider incorporating tau biomarkers for enhanced patient stratification. Second, the cross‐sectional design is vulnerable to various confounding factors, contributing to significant inter‐individual variability in neurometabolism and neuropsychological performance. Longitudinal studies would better facilitate a direct examination of the hypothesis linking neurometabolic changes with cognitive decline. Specifically, enrolling participants at pre‐symptomatic stages may capture the early emergence of both neurometabolic changes and cognitive symptoms. Additionally, our study exclusively focused on an East Asian population. Future research should include more diverse samples to rigorously explore potential racial/ethnic differences and enhance the generalizability of findings. Finally, while our study provides valuable insights into the impact of AD on the neurometabolic levels, it is essential to acknowledge the limitations posed by overlapping group differences due to the heterogeneity among AD patients. This renders our current results underpowered, given the constraints of our limited sample size. Consequently, the identification of predictive features requires further investigation with a larger sample size and finer feature extraction to enhance the discriminatory power between healthy and patient groups. Furthermore, recent studies using deep learning or other machine learning methods have demonstrated promising results in predicting cognitive functions.^75^ There is considerable potential for further advancements in predicting cognitive function using whole‐brain neurometabolic levels through the integration of these techniques.

## 5 CONCLUSION

In this study, we evaluated the neurometabolic topography and its associations with cognition in Aβ‐positive patients with AD clinical syndrome, compared with a group of Aβ‐negative controls, utilizing a novel whole‐brain high‐resolution MRSI technology. We demonstrated the clinical relevance of NAA reduction, progressing from the limbic system to the superior temporal cortex, as well as mI increase, initially in the precuneus then progressing to parietal and temporal regions in the development of AD. Cognitive function was primarily associated with NAA reduction, with visual episodic memory primarily correlated with temporo‐parietal regions, while verbal episodic memory associated with both temporo‐parietal and limbic regions. Our results unveiled the potential of neurometabolic topography in predicting clinical status and cognitive deficits in AD. Future studies will continue to evaluate the utility of whole‐brain high‐resolution MRSI for tracking AD progression and monitoring potential therapies.

## CONFLICT OF INTEREST STATEMENT

The authors declare no conflict of interest pertinent to the current study. Author disclosures are available in the supporting information.

## CONSENT STATEMENT

Written informed consent was obtained from all participants in this study.

## Supporting information

Supporting Information

Supporting Information
