# Supplementary material for: Neurometabolic topography and associations with cognition in Alzheimer's disease: A whole‐brain high‐resolution 3D MRSI study
Source: Alzheimers Dement. 2024 Jul 29;20(9):6407–22. doi: 10.1002/alz.14137 (PMC11497670; doi:10.1002/alz.14137)
Supplement: Supplementary file 1 — Supporting Information [file ALZ-20-6407-s002.docx]

**Supplementary Materials**

**Supplementary Figure 1**

**
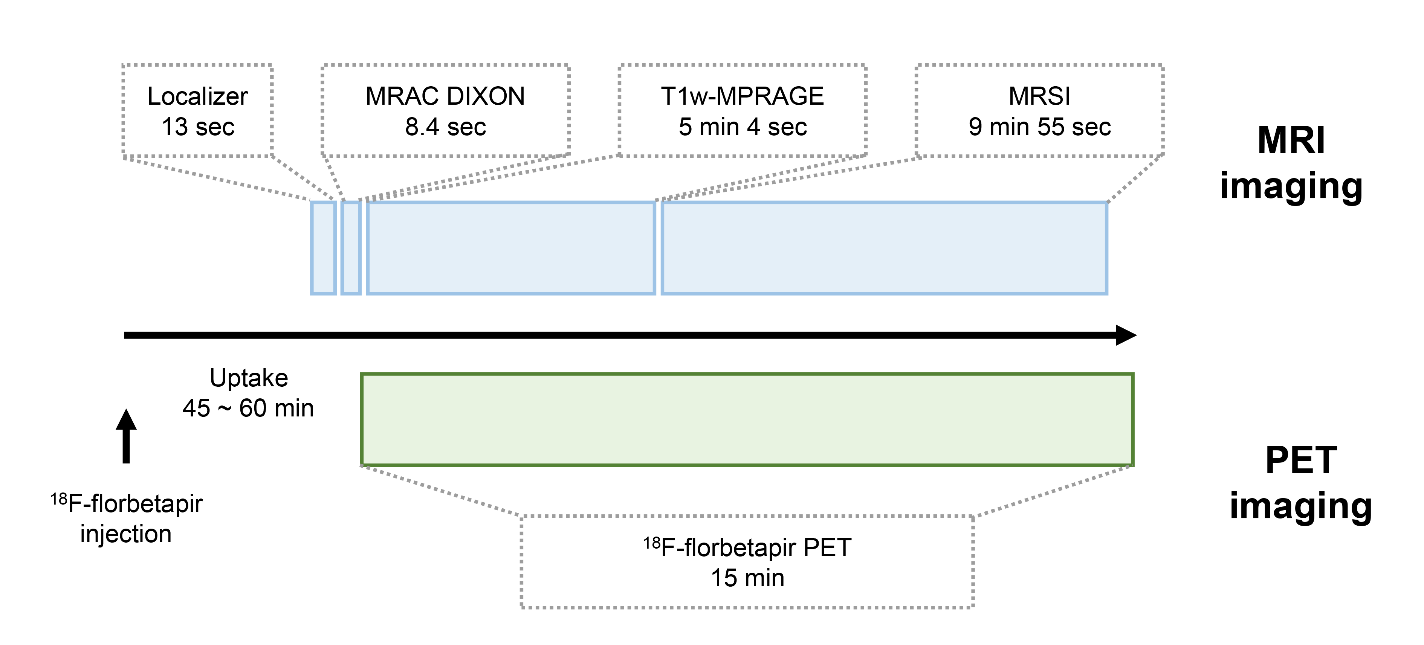
**

Schematic diagram of the simultaneously acquired ^18^F-florbetapir PET/MRI/MRSI protocol. Abbreviations: PET, positron emission tomography; MRI, magnetic resonance imaging; MRSI, magnetic resonance spectroscopic imaging; MPRAGE, magnetization-prepared rapid gradient echo.

**Supplementary Figure 2**

**
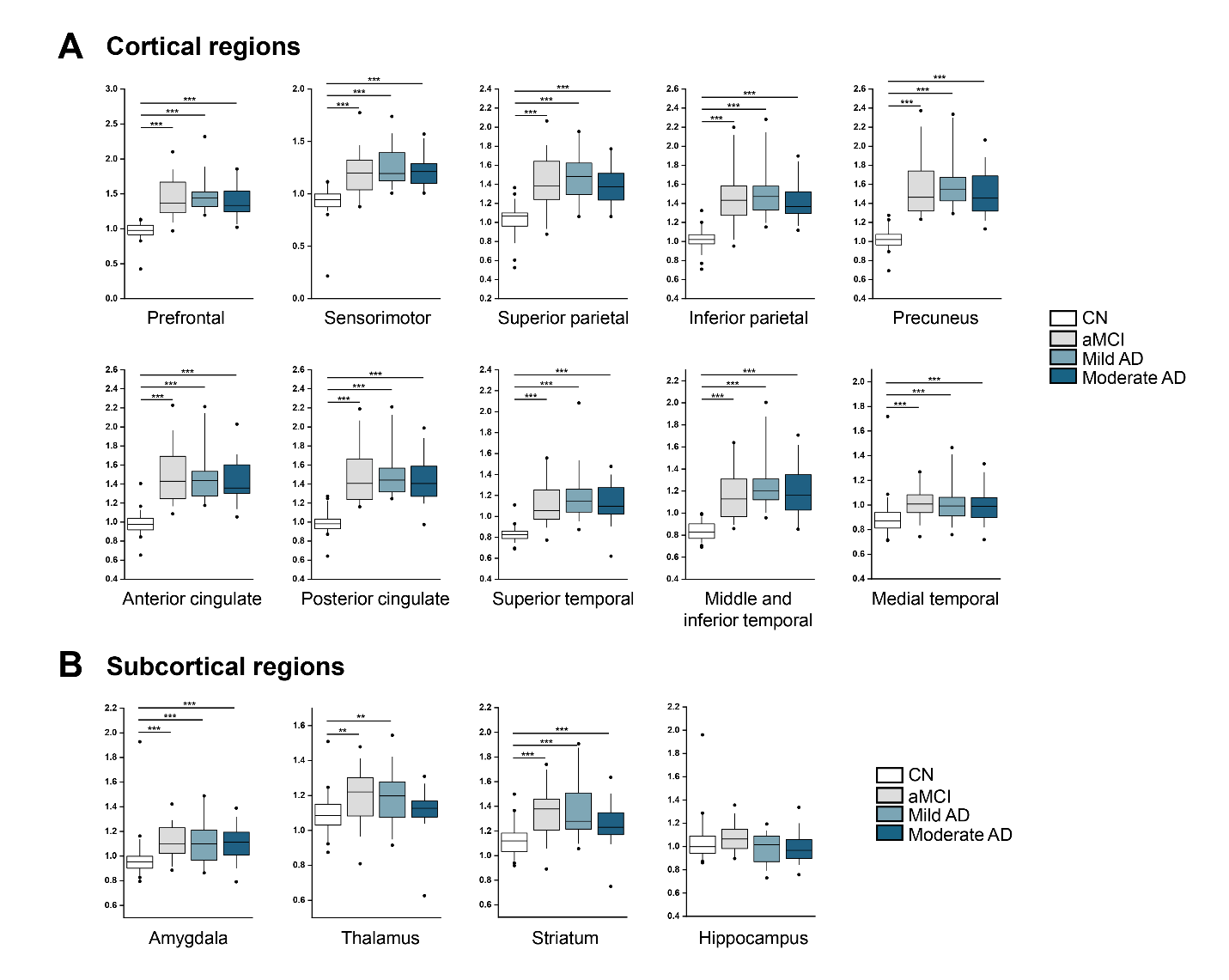
**

Comparisons of amyloid-β SUVR values between CN, aMCI, mild AD and moderate AD groups in (A) cortical and (B) subcortical regions, respectively. Boxplots display mean ± interquartile range, with whiskers representing the 5-95% percentiles. P values from post hoc pairwise comparisons were Bonferroni-corrected. *P < 0.05; **P < 0.01; ***P < 0.001. Abbreviations: SUVR, standard uptake value ratio; CN, cognitively normal; aMCI, amnestic mild cognitive impairment; AD, Alzheimer’s disease.

**Supplementary Figure 3**

**
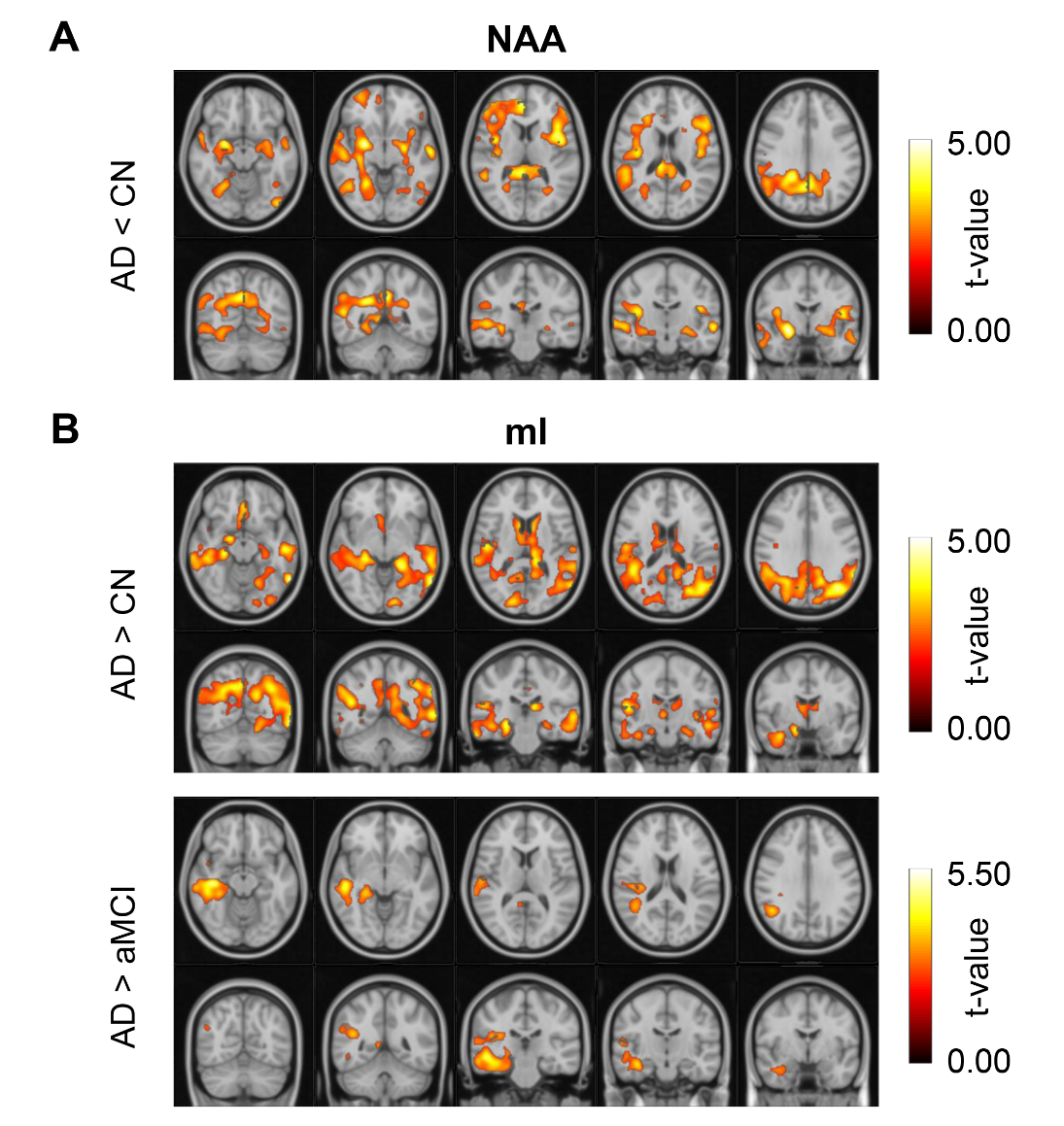
**

Voxel-wise comparisons of neurometabolic levels between groups. Clusters display the t-values for the significant results of (A) differences in NAA level between AD and CN and (B) differences in mI level between AD and CN or between AD and aMCI. These results were overlaid onto a T1-weighted MNI template for visualization. Abbreviations: NAA, N-acetylaspartate; mI, myo-inositol; AD, Alzheimer’s disease; CN, cognitively normal; aMCI, amnestic mild cognitive impairment; MNI, Montreal Neurological Institute.

**Supplementary Figure 4**

**
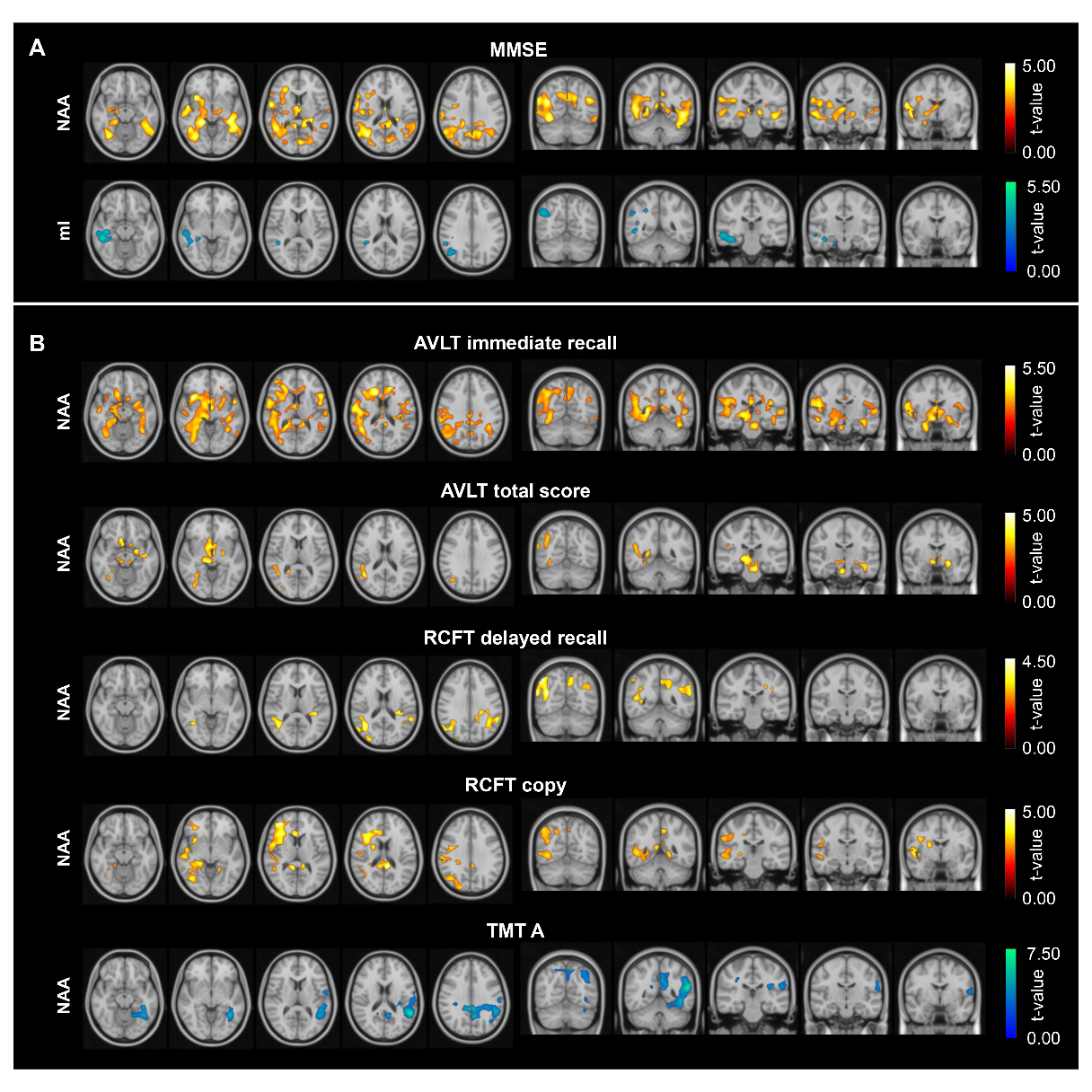
**

Voxel-wise correlations of neurometabolic levels with cognitive measures in prodromal AD and AD dementia patients. (A) Clusters display the t-values for the significant results of NAA and mI with MMSE. (B) Clusters display the t-values for the significant results of NAA with specific neuropsychological measures. These results were overlaid onto a T1-weighted MNI template for visualization. Hot and winter colors indicate positive and negative associations, respectively. Abbreviations: NAA, N-acetylaspartate; mI, myo-inositol; aMCI, amnestic mild cognitive impairment; AD, Alzheimer’s disease; MMSE, Mini-Mental State Examination; AVLT, Auditory Verbal Learning Test; TMT A, Trail Making Test Parts A; MNI, Montreal Neurological Institute.

**Supplementary Table 1.** Regional choline and creatine levels in amyloid-β positive patients compared with amyloid-β negative controls.

| **Regions of interest** | **Cho** | | |  | **Cr** | | |
| --- | --- | --- | --- | --- | --- | --- | --- |
|  | Aβ+ patients | Aβ- controls | P |  | Aβ+ patients | Aβ- controls | P |
| Prefrontal | 0.015 ± 0.001 | 0.016 ± 0.001 | 0.20 |  | 0.022 ± 0.002 | 0.022 ± 0.001 | 0.86 |
| Sensorimotor | 0.015 ± 0.001 | 0.015 ± 0.001 | 0.20 |  | 0.021 ± 0.001 | 0.021 ± 0.001 | 0.86 |
| Superior parietal | 0.013 ± 0.001 | 0.013 ± 0.001 | 0.26 |  | 0.021 ± 0.002 | 0.021 ± 0.002 | 0.79 |
| Inferior parietal | 0.013 ± 0.001 | 0.013 ± 0.001 | 0.093 |  | 0.020 ± 0.002 | 0.020 ± 0.002 | 0.86 |
| Precuneus | 0.015 ± 0.001 | 0.015 ± 0.001 | 0.73 |  | 0.023 ± 0.002 | 0.022 ± 0.002 | 0.79 |
| Anterior cingulate | 0.021 ± 0.002 | 0.021 ± 0.003 | 0.73 |  | 0.028 ± 0.002 | 0.028 ± 0.002 | 0.79 |
| Posterior cingulate | 0.016 ± 0.002 | 0.017 ± 0.002 | 0.33 |  | 0.023 ± 0.002 | 0.023 ± 0.001 | 0.79 |
| Superior temporal | 0.015 ± 0.001 | 0.016 ± 0.001 | 0.090 |  | 0.021 ± 0.001 | 0.021 ± 0.001 | 0.86 |
| Middle and inferior temporal | 0.012 ± 0.001 | 0.012 ± 0.001 | 0.26 |  | 0.017 ± 0.001 | 0.017 ± 0.001 | 0.79 |
| Medial temporal | 0.016 ± 0.002 | 0.016 ± 0.001 | 0.41 |  | 0.026 ± 0.002 | 0.026 ± 0.002 | 0.79 |
| Hippocampus | 0.020 ± 0.002 | 0.020 ± 0.002 | 0.090 |  | 0.020 ± 0.001 | 0.020 ± 0.002 | 0.86 |
| Amygdala | 0.022 ± 0.003 | 0.023 ± 0.003 | 0.060 |  | 0.021 ± 0.001 | 0.021 ± 0.001 | 0.79 |
| Thalamus | 0.018 ± 0.002 | 0.019 ± 0.001 | 0.31 |  | 0.023 ± 0.002 | 0.022 ± 0.002 | 0.79 |
| Striatum | 0.018 ± 0.002 | 0.018 ± 0.002 | 0.20 |  | 0.024 ± 0.002 | 0.024 ± 0.001 | 0.86 |
| Fornix | 0.018 ± 0.002 | 0.019 ± 0.002 | 0.090 |  | 0.023 ± 0.002 | 0.023 ± 0.001 | 0.79 |

Abbreviations: Cho, choline; Cr, creatine; Aβ+, amyloid-β positive; Aβ-, amyloid-β negative.

Data are presented as mean ± SD. P values are obtained from analysis of covariance conducted between Aβ+ patients and Aβ- controls, with age, sex, and education years as covariates. All P values are false discovery rate-corrected for multiple comparisons for each neurometabolic level.

**Supplementary Table 2.** Associations between regional Aβ SUVR values and cognitive measures in prodromal AD and AD dementia patients

| **Aβ SUVR** | **MMSE** | | |  | **AVLT immediate recall** | | |  | **AVLT total score** | | |  | **RCFT delayed recall** | | |  | **RCFT copy** | | |  | **TMT A** | | |
| --- | --- | --- | --- | --- | --- | --- | --- | --- | --- | --- | --- | --- | --- | --- | --- | --- | --- | --- | --- | --- | --- | --- | --- |
|  | B | 95% CI | P |  | B | 95% CI | P |  | B | 95% CI | P |  | B | 95% CI | P |  | B | 95% CI | P |  | B | 95% CI | P |
| Prefrontal | -0.03 | [-0.26, 0.20] | 0.79 |  | 0.03 | [-0.37, 0.43] | 0.89 |  | -0.16 | [-0.60, 0.29] | 0.48 |  | 0.16 | [-0.19, 0.51] | 0.37 |  | 0.00 | [-0.35, 0.36] | 0.98 |  | 0.05 | [-0.42, 0.52] | 0.83 |
| Sensorimotor | -0.14 | [-0.37, 0.09] | 0.22 |  | -0.16 | [-0.57, 0.25] | 0.44 |  | -0.19 | [-0.64, 0.26] | 0.39 |  | 0.14 | [-0.23, 0.50] | 0.45 |  | -0.21 | [-0.57, 0.15] | 0.25 |  | 0.19 | [-0.28, 0.65] | 0.41 |
| Superior parietal | -0.01 | [-0.24, 0.22] | 0.94 |  | 0.00 | [-0.40, 0.4] | 0.99 |  | -0.05 | [-0.48, 0.38] | 0.81 |  | 0.14 | [-0.20, 0.49] | 0.41 |  | -0.01 | [-0.37, 0.34] | 0.95 |  | 0.04 | [-0.40, 0.48] | 0.85 |
| Inferior parietal | 0.05 | [-0.18, 0.28] | 0.67 |  | 0.14 | [-0.25, 0.53] | 0.46 |  | 0.02 | [-0.42, 0.46] | 0.94 |  | 0.16 | [-0.19, 0.51] | 0.35 |  | 0.05 | [-0.31, 0.40] | 0.80 |  | 0.03 | [-0.42, 0.47] | 0.90 |
| Precuneus | 0.01 | [-0.22, 0.24] | 0.92 |  | 0.06 | [-0.32, 0.44] | 0.74 |  | -0.05 | [-0.47, 0.37] | 0.81 |  | 0.21 | [-0.12, 0.54] | 0.20 |  | 0.05 | [-0.29, 0.39] | 0.77 |  | 0.02 | [-0.40, 0.45] | 0.91 |
| Anterior cingulate | 0.01 | [-0.22, 0.24] | 0.93 |  | 0.11 | [-0.28, 0.49] | 0.58 |  | -0.07 | [-0.50, 0.36] | 0.74 |  | 0.17 | [-0.17, 0.51] | 0.31 |  | 0.05 | [-0.30, 0.39] | 0.79 |  | -0.14 | [-0.58, 0.30] | 0.51 |
| Posterior cingulate | 0.03 | [-0.19, 0.26] | 0.76 |  | 0.09 | [-0.29, 0.47] | 0.65 |  | -0.07 | [-0.50, 0.35] | 0.72 |  | 0.22 | [-0.11, 0.55] | 0.18 |  | 0.06 | [-0.28, 0.40] | 0.71 |  | -0.01 | [-0.45, 0.43] | 0.96 |
| Superior temporal | -0.02 | [-0.25, 0.21] | 0.85 |  | 0.06 | [-0.35, 0.46] | 0.78 |  | -0.16 | [-0.63, 0.31] | 0.50 |  | 0.12 | [-0.23, 0.47] | 0.49 |  | -0.04 | [-0.40, 0.31] | 0.81 |  | 0.16 | [-0.33, 0.65] | 0.50 |
| Middle and inferior temporal | -0.07 | [-0.30, 0.16] | 0.53 |  | 0.11 | [-0.28, 0.5] | 0.57 |  | -0.01 | [-0.44, 0.43] | 0.98 |  | 0.02 | [-0.33, 0.37] | 0.92 |  | -0.03 | [-0.39, 0.32] | 0.85 |  | 0.14 | [-0.30, 0.58] | 0.51 |
| Medial temporal | 0.00 | [-0.23, 0.22] | 0.97 |  | 0.05 | [-0.35, 0.44] | 0.81 |  | 0.03 | [-0.39, 0.46] | 0.88 |  | 0.12 | [-0.21, 0.46] | 0.45 |  | 0.02 | [-0.32, 0.36] | 0.92 |  | -0.09 | [-0.51, 0.33] | 0.65 |
| Hippocampus | 0.21 | [-0.01, 0.43] | 0.057^*^ |  | 0.26 | [-0.10, 0.63] | 0.15 |  | 0.23 | [-0.17, 0.63] | 0.24 |  | 0.24 | [-0.08, 0.56] | 0.13 |  | 0.06 | [-0.27, 0.40] | 0.71 |  | -0.39 | [-0.77, -0.01] | **0.046** |
| Amygdala | -0.01 | [-0.24, 0.21] | 0.92 |  | 0.11 | [-0.26, 0.48] | 0.56 |  | -0.02 | [-0.43, 0.39] | 0.91 |  | 0.13 | [-0.20, 0.45] | 0.43 |  | 0.03 | [-0.30, 0.36] | 0.86 |  | -0.09 | [-0.50, 0.32] | 0.66 |
| Thalamus | 0.17 | [-0.06, 0.39] | 0.14 |  | 0.10 | [-0.28, 0.48] | 0.58 |  | -0.05 | [-0.48, 0.38] | 0.80 |  | 0.27 | [-0.04, 0.59] | 0.088 |  | 0.28 | [-0.04, 0.60] | 0.083 |  | -0.40 | [-0.81, 0.01] | 0.055 |
| Striatum | 0.12 | [-0.11, 0.35] | 0.30 |  | 0.19 | [-0.19, 0.56] | 0.31 |  | -0.05 | [-0.49, 0.40] | 0.83 |  | 0.27 | [-0.05, 0.59] | 0.095 |  | 0.23 | [-0.10, 0.56] | 0.17 |  | -0.20 | [-0.65, 0.25] | 0.37 |

Abbreviations: Aβ, amyloid-β; SUVR, standardized uptake value ratio; MMSE, Mini-Mental State Examination; AVLT, Auditory Verbal Learning Test; RCFT, Rey-Osterrieth Complex Figure Test; TMT A, Trail Making Test Parts A; AD, Alzheimer’s disease.

Standardized coefficients (B), 95% confidence intervals (95% CI) and P values derived from linear regression models are reported. In the linear regression model, cognitive measure was regressed on the regional Aβ SUVR value, adjusting for age, sex and education years. Significant P values are in bold.

* P < 0.05 when adjusting the models for total gray matter volume.
